# Obesity-Linked Homologues *TfAP-2* and *Twz* Establish Meal Frequency in *Drosophila melanogaster*

**DOI:** 10.1371/journal.pgen.1004499

**Published:** 2014-09-04

**Authors:** Michael J. Williams, Philip Goergen, Jayasimman Rajendran, Galina Zheleznyakova, Maria G. Hägglund, Emelie Perland, Sonchita Bagchi, Argyro Kalogeropoulou, Zaid Khan, Robert Fredriksson, Helgi B. Schiöth

**Affiliations:** Functional Pharmacology, Department of Neuroscience, Uppsala University, Uppsala, Sweden; The University of North Carolina at Chapel Hill, United States of America

## Abstract

In all animals managing the size of individual meals and frequency of feeding is crucial for metabolic homeostasis. In the current study we demonstrate that the noradrenalin analogue octopamine and the *cholecystokinin* (*CCK*) homologue *Drosulfakinin* (*Dsk*) function downstream of *TfAP-2* and *Tiwaz* (*Twz*) to control the number of meals in adult flies. Loss of *TfAP-2* or *Twz* in octopaminergic neurons increased the size of individual meals, while overexpression of *TfAP-2* significantly decreased meal size and increased feeding frequency. Of note, our study reveals that *TfAP-2* and *Twz* regulate octopamine signaling to initiate feeding; then octopamine, in a negative feedback loop, induces expression of *Dsk* to inhibit consummatory behavior. Intriguingly, we found that the mouse *TfAP-2* and *Twz* homologues, AP-2β and Kctd15, co-localize in areas of the brain known to regulate feeding behavior and reward, and a proximity ligation assay (PLA) demonstrated that AP-2β and Kctd15 interact directly in a mouse hypothalamus-derived cell line. Finally, we show that in this mouse hypothalamic cell line AP-2β and Kctd15 directly interact with Ube2i, a mouse sumoylation enzyme, and that AP-2β may itself be sumoylated. Our study reveals how two obesity-linked homologues regulate metabolic homeostasis by modulating consummatory behavior.

## Author Summary

The size of individual meals and feeding frequency are important for homeostatic control. Due to the complex neuroendocrine system regulating human food intake it is difficult to uncover the mechanisms underlying eating disorders. The genetically tractable model system *Drosophila melanogaster* has a comparatively simple brain; yet, similar to humans, its eating behavior can adapt to respond to nutritional needs. Our study describes how the obesity-linked homologues *TfAP-2* (human *TFAP2B*) and *Tiwaz* (human *KCTD15*) regulate a unique feedback system involving noradrenalin-like octopamine and the *CCK* homolog *Dsk*, that exert positive and negative effects on *Drosophila* feeding behavior. Our findings provide insight into how two conserved obesity-linked genes regulate feeding behavior in order to maintain metabolic balance.

## Introduction

The human genes *TFAP2B* (encoding AP-2β) and *KCTD15* were strongly linked to obesity in multiple genome-wide association studies (GWAS) [1]–[4], though it is still not understood how they regulate obesity. In the fruit fly *Drosophila melanogaster, TFAP2B* and *KCTD15* are both highly conserved, encoded by *TfAP-2* and *Tiwaz* (*Twz*), respectively [5]–[7]. Recently, it was shown in zebrafish embryos that Kctd15 interacts directly with AP-2α (*Tfap2a*) to inhibit AP-2α function, and in *Drosophila* there is evidence for an association between *TfAP-2* and *Twz* [6], [8], [9]. TfAP-2 and Twz had a strong interaction in a large scale yeast two-hybrid screen using almost the entire *Drosophila* proteome [9]. Moreover, we have shown in adult males that *TfAP-2* and *Twz* genetically interact to control aggressive behavior by regulating octopamine production and secretion, which in turn regulates the expression of the *Drosophila cholecystokinin* (*CCK*) homolog *Drosulfakinin* (*Dsk*) [6]. Interestingly, we demonstrated that overexpression of *Dsk* was sufficient to induce aggressive behavior in males.

CCK, a mammalian gastrointestinal hormone, is secreted by the gut when nutrients enter the lumen. After being released CCK binds to the cholecystokinin A receptor (CCKAR) located on vagal sensory terminals, this pathway delivers satiation signals to the nucleus of the solitary tract (NTS) to inhibit feeding [10], [11]. Similar to mammalian CCK, in *Drosophila* adults *Dsk* is necessary to inhibit overeating after starvation [12]. Furthermore, it was reported that *Dsk* is necessary in larvae and adult *Drosophila* to determine food palatability [12]. More recently, it was determined that octopamine also has an important role in determining the palatability of food [13]. These results led us to ask the following questions: Are *TfAP-2* and *Twz* involved in regulating *Drosophila* adult feeding behavior? Does *Dsk* regulate normal *ab lib* feeding behavior? Do octopamine and Dsk interact to regulate feeding in adult flies?

Here, using genetic tools to manipulate their expression, we have investigated the function of *TfAP-2* and *Twz* in the regulation of feeding behavior. Our data suggest that *TfAP-2* and *Twz* control feeding through octopamine signaling. Furthermore, we demonstrate that octopamine and Dsk interact in a negative feedback loop to control the frequency of meals. Moreover, this function may be conserved in mammals, as we discovered that mouse AP-2β and Kctd15 proteins directly interact in a mouse hypothalamic cell line and co-localize in areas of the mouse brain involved in modulating feeding behavior. Finally, we demonstrate that similar to other members of their protein families both AP-2β and Kctd15 interact directly with the sumoylation enzyme Ube2i.

## Results

### Starvation and diet affect *TfAP-2* and *Twz* transcription

**Figure 1:**
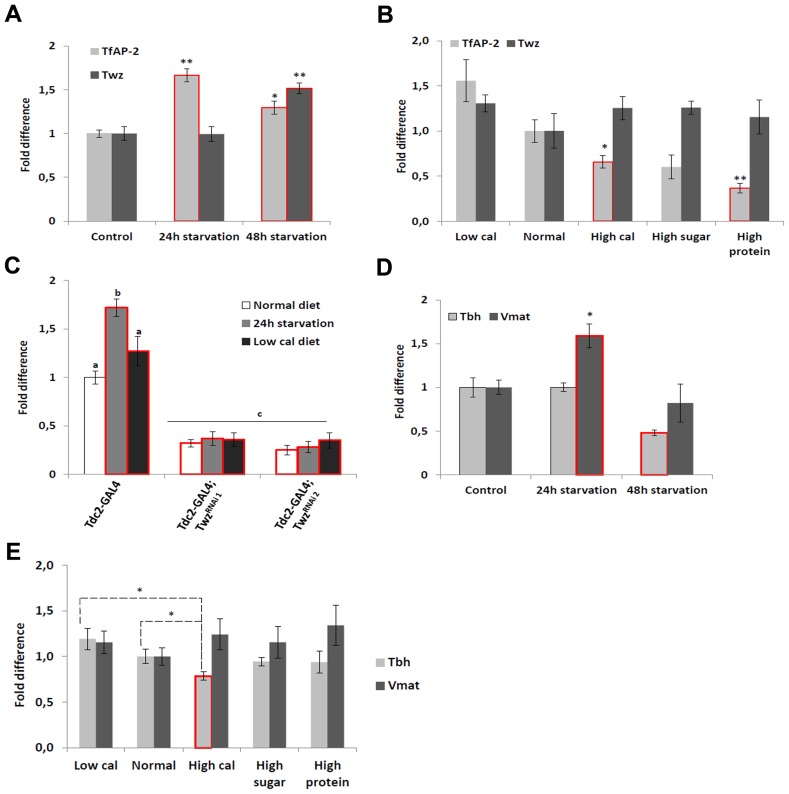
Diet regulates *TfAP-2* and *Twz* transcript levels. (A) Relative levels of *Tfap-2* or *Twz* transcript in the heads of flies starved either for 24 or 48 h. (B) Relative levels of *Tfap-2* or *Twz* transcript in the heads of flies kept on various diets for 5 days. (A–B) RNA was collected from the heads of 50, 5–7 day old, males for each genotype. qPCR was repeated at least 7 times for each transcript. (C) Relative levels of *Tfap-2* transcript in the heads of control and *Twz* knockdown flies starved either for 24 h or kept on a low calorie diet (2.5 g·dl^−1^∶2.5 g·dl^−1^ sucrose∶brewer's yeast) for 5 days. RNA was collected from the heads of 50, 5–7 day old, males for each genotype. qPCR was repeated at least 7 times for each transcript. (D) Relative levels of *Tbh* or *Vmat* transcript in the heads of flies starved either for 24 or 48 h. (E) Relative levels of *Tbh* or *Vmat* transcript in the heads of flies kept on various diets for 5 days. (For all assays n = 10 qPCR runs; Different letters indicate similar groups (i.e. ‘a’ is significantly different than ‘b’ or ‘c’ and so on, one-way ANOVA with Bonferroni post hoc test for multiple comparisons, P<0.05). A single asterisk indicates significant difference in two-way ANOVA, P<0.05, while a double asterisk indicates significant difference in two-way ANOVA, P<0.005). Error bars represent the SE (SEM).

Previously, we demonstrated that *TfAP-2* and *Twz* function in octopaminergic neurons to regulate the expression of the *Drosophila cholecystokinin* (*CCK*) homologue *Drosulfakinin* (*Dsk*), via octopamine signaling [6]. It was reported that *Dsk* is involved in regulating consummatory behavior [12], to understand if *TfAP-2* and *Twz* could also be involved in commsumatory behavior we performed qPCR to determine their transcript levels after different dietary regiments. Intriguingly, compared to flies fed *ab lib*, starving males for 24 h significantly increased *TfAP-2* expression (1.66-fold, SE ±0.07, P<0.005) (Figure 1A), but not *Twz* expression. Starving the males for 48 h significantly increased the expression of both genes (Figure 1A). Next, we determined if macronutrient content influenced *TfAP-2* and *Twz* expression. The transcript levels of *TfAP-2* and *Twz* in males fed a normal diet (10 g·dl^−1^∶10 g·dl^−1^ sucrose∶brewer's yeast S:Y) were set as 100%, represented as 1 on the graph (Figure 1B). Keeping males for 5 days on a the low calorie diet (2.5 g·dl^−1^∶2.5 g·dl^−1^ S:Y) increased the expression of *TfAP-2*, but not significantly (1.56-fold, SE ±0.23, P = 0.056). Feeding males a high calorie diet (40 g·dl^−1^∶40 g·dl^−1^ S:Y) or just a high protein diet (10 g·dl^−1^∶40 g·dl^−1^ S:Y) significantly reduced the expression of *TfAP-2* (*high calorie diet*: 0.66-fold SE ±0.07, P<0.05; *high protein diet*: 0.37-fold, SE ±0.05, P<0.005). The high sugar diet (40 g·dl^−1^∶10 g·dl^−1^ S:Y) also decreased *TfAP-2* expression, but not significantly (0.60-fold, SE ±1.26, P = 0.059) (Figure 1B). None of the diets had a considerable effect on *Twz* expression (Figure 1B). From these results it is evident that *TfAP-2* transcript levels are up-regulated under conditions of dietary restriction and down-regulated when flies are fed a high calorie diet; while *Twz* transcription is only influenced by severe starvation.

We already established that *Twz* was necessary for *TfAP-2* expression in octopaminergic neurons (Text S1, Figure S1A) [6]. To understand if *Twz* was necessary for the increase observed in *TfAP-2* expression when flies were starved or raised on a low calorie diet, we knocked down *Twz* in octopaminergic neurons and performed qPCR analysis. The transcript levels of *TfAP-2* in males fed *ab lib* on a normal diet (10 g·dl^−1^∶10 g·dl^−1^ S:Y) was set as 100%, represented as 1 on the graph (Figure 1C). As seen previously, starving control males (*Tdc2-GAL4^+/−^*) for 24 h significantly increased *TfAP-2* expression (1.72-fold, SE ±0.07, P<0.005), whereas keeping them for 5 days on a low calorie diet (2.5 g·dl^−1^∶2.5 g·dl^−1^ S:Y) also increased *TfAP*-2 levels, but not significantly (Figure 1C). Interestingly, knocking down *Twz* expression in octopaminergic neurons was sufficient to significantly inhibit *TfAP-2* expression in starved flies, as well as flies raised on a low calorie diet (Figure 1C).

Previously we have reported that overexpression of *TfAP-2* in octopaminergic neurons is sufficient to induce the expression of two gene necessary for octopamine production and secretion, *Tyramine β hydroxylase* (*Tbh*) and *Vesicular monoamine transporter* (*Vmat*) [6]. Since starvation had a significant effect on *TfAP-2* transcription, to understand if starvation influenced *Tbh* and *Vmat* transcriptional levels we performed qPCR analysis on adult males starved for 24 h or 48 h. Intriguingly, compared to flies fed *ab lib*, starving males for 24 h significantly increased *Vmat* expression (1.59-fold, SE ±0.11, P<0.05) (Figure 1D). By 48 h of starvation *Vmat* expression was back to control levels. Starving males for 48 h significantly decreased *Tbh* expression levels (0.48-fold, SE ±0.03, P<0.05) compared to controls. Next, we determined if macronutrient content influenced *Tbh* or *Vmat* expression. While none of the diets had a considerable effect on *Vmat* expression, a slight increase in *Tbh* expression was observed in flies fed a low calorie diet, and a significant decrease was observed in flies fed a high calorie diet (0.79, SE ±0.05, P<0.05) (Figure 1E). From these results it appears that starvation conditions regulate both *Tbh* and *Vmat* transcription, but only *Tbh* is regulated by macronutrient content.

### *TfAP-2* and *Twz* regulate feeding after starvation

**Figure 2:**
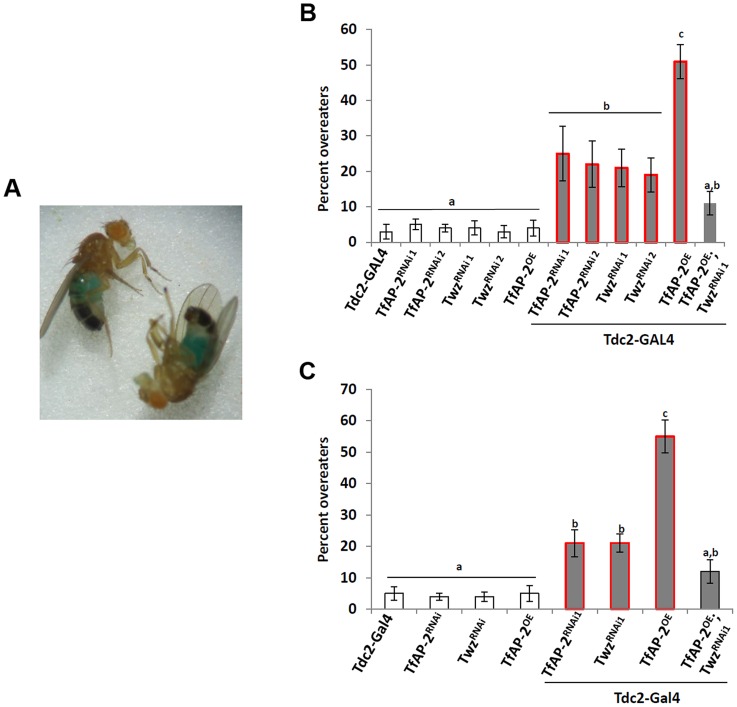
*TfAP-2* and *Twz* regulate feeding behavior after starvation. (A) Ingested dyed-food was visually detected in the abdomen of adult males. (B and B_1_) After being starved for 16 h male flies were first fed normal food (5% sucrose, 5% yeast and 1% agarose) for 20 minutes, then switched to normal food containing (B) 2% blue food coloring or (C) 2% green food coloring for 10 minutes. After this time the number of flies with blue guts was counted. This assay was repeated at least 5 times with 20 males used for each replicate. (B and C: Different letters indicate similar groups (i.e. ‘a’ is significantly different from ‘b’ or ‘c’ and so on, one-way ANOVA with Bonferroni post hoc test for multiple comparisons, P<0.05). Error bars represent the SE (SEM).

The *Drosophila CCK* homologue *Dsk* is necessary to limit meal size after starvation [12], and we saw that both *TfAP-2* and *Twz* transcript levels were increased under starvation conditions (Figure 1A). To discover if *TfAP-2* and *Twz* could be involved in regulating consummatory behavior after starvation we performed a re-feeding assay. Normally flies re-introduced to food after overnight starvation will eat for ∼20 minutes [14]. We allowed starved flies to feed on normal food for 20 minutes before letting them feed for a further 10 minutes on normal food containing blue food dye. Overeaters were determined as those males having blue food in their guts when observed under a stereomicroscope [12], [14] (Figure 2A). Only 3% (SE ±2) of *Tdc2-GAL4^+/−^* control flies ate the colored food, indicating that most control flies were satiated after 20 minutes, while 25% (SE ±7.7, P<0.05) of *TfAP-2^RNAi1^* and 22% (SE ±6.5, P<0.05) of *TfAP-2^RNAi2^* flies overate; 21% (SE ±5.3, P<0.05) of *Twz^RNAi1^* and 19% (SE ±4.8, P<0.05) of *Twz^RNAi2^* males overate (Figure 2B). Intriguingly, 50% (SE ±4.8, P<0.005) of *TfAP-2^OE^* males overate after being starved for 16 h, while only 10% (SE ±4.2, P<0.05) of the *TfAP-2^OE^*;*Twz^RNAi1^* flies continued to eat beyond the initial 20 minutes (Figure 2B). Similar results were obtained using green food dye; to exclude any possible effects caused by the blue food coloring (Figure 2C). Interestingly, similar to loss of *Dsk* in the insulin-producing cells, knocking down *Tfap-2* or *Twz* in octopaminergic neurons causes them to overeat after starvation, indicating they may be upstream of *Dsk* in regulating consummatory behavior. Of note, when *Tfap-2* was overexpressed in octopaminergic neurons an even stronger overeating phenotype was observed than with loss of *Tfap-2*.

### *TfAP-2* and *Twz* are necessary for proper consummatory behavior

**Figure 3:**
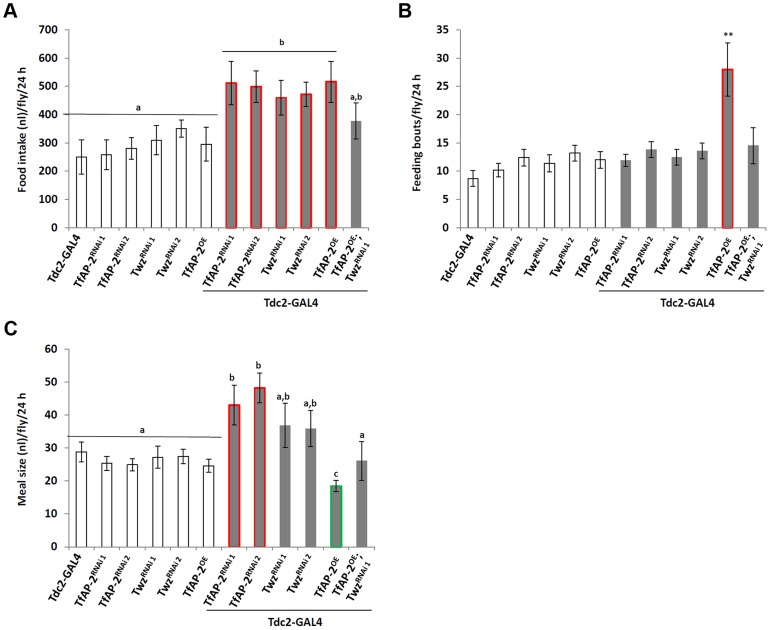
*TfAP-2* and *Twz* regulate normal feeding behavior. (A–C) A CAFE assay was used to assess total food intake (A), the average number of feeding bouts (B) and meal size (C) over a 24 h period in 5–7 days old adult males. Five males were used for each replicate and the assay was repeated at least 10 times for each genotype. In A and C, different letters indicate similar groups (i.e. ‘a’ is significantly different than ‘b’ or ‘c’ and so on. A non-parametric Kruskal-Wallis ANOVA was performed with Dunn's post hoc test for multiple comparisons, P<0.05), in B a double asterisk indicates significant difference (A non-parametric Kruskal-Wallis ANOVA was performed with Dunn's post hoc test for multiple comparisons, P<0.005) compared with all other strains. Error bars represent the SE (SEM).

To understand if *TfAP-2* and *Twz* were involved in regulating normal consummatory behavior in adults, a CAFE assay was performed to measure how much food flies fed *ad lib* consumed during a 24 h period [15]. While *Tdc2-GAL4^+/−^* control flies ate 270 nl (SE ±60.9), *TfAP-2^RNAi1^* and *TfAP2^RNAi2^* knockdown flies ate significantly more, 511.8 nl (SE ±76.7, P<0.005) and 499.3 nl (SE ±61.1, P<0.05), respectively (Figure 3A). Similar results were obtained when we knocked down *Twz* (*Twz^RNAi1^*: 460.2 nl, SE ±61.1, P<0.005; *Twz^RNAi2^*: 472 nl, SE ±43.3, P<0.005). Of note, overexpressing *TfAP-2* specifically in octopaminergic neurons also induced flies to eat significantly more than controls (490 nl, SE ±73, P<0.005) (Figure 3A). The increase in meal size due to *TfAP-2* overexpression was rescued by simultaneously knocking down *Twz* (*TfAP2^OE^;Twz^RNAi1^*: 290 nl, SE ±63.3, P = 0.86).

To observe each genotype's consummatory behavior, individual CAFE assays were videoed, allowing us to determine both the number of feeding bouts (fb) and the average meal size. Controls had 9 fb per fly (SE ±1.1) over a 24 h period, while flies overexpressing *TfAP-2* in octopaminergic neurons had 28 fb (SE ±5.1, P<0.005) (Figure 3B). Compared to *TfAP-2^OE^* males, *TfAP-2^OE^;Twz^RNAi1^* males had significantly fewer feeding bouts per fly (14 fb, SE ±3.2, P<0.005). Meal size was also affected by the various genotypes. Control flies ate on average 29 nl per meal (SE ±3.0), while *TfAP-2^RNAi1^* and *TfAP-2^RNAi2^* knockdown males ate significantly larger meals, 43 nl (SE ±6.0, P<0.05) and 48.2 nl (SE ±4.5, P<0.05), respectively. *Twz^RNAi1^* and *Twz^RNAi2^* males ate slightly, but not significantly, larger meals (*Twz^RNAi1^* ^:^ 36.8 nl, SE ±6.7, P = 0.3; *Twz^RNAi2^*: 35.9 nl, SE ±5.5) (Figure 3C). Overexpression of *TfAP-2* in octopaminergic neurons induced a significant reduction in average meal size (18 nl, SE ±2.0, P<0.005) (Figure 3C), which was rescued by simultaneously knocking down *Twz* (*TfAP2^OE^;Twz^RNAi1^*: 26 nl, SE ±6.0, P = 0.68). The increase in feeding bouts observed when *TfAP-2* was overexpressed in octopaminergic neurons might explain overeating after starvation, since overexpression of *TfAP-2* induced flies to feed.

### *TfAP-2* and *Twz* regulate feeding through octopamine signaling

**Figure 4:**
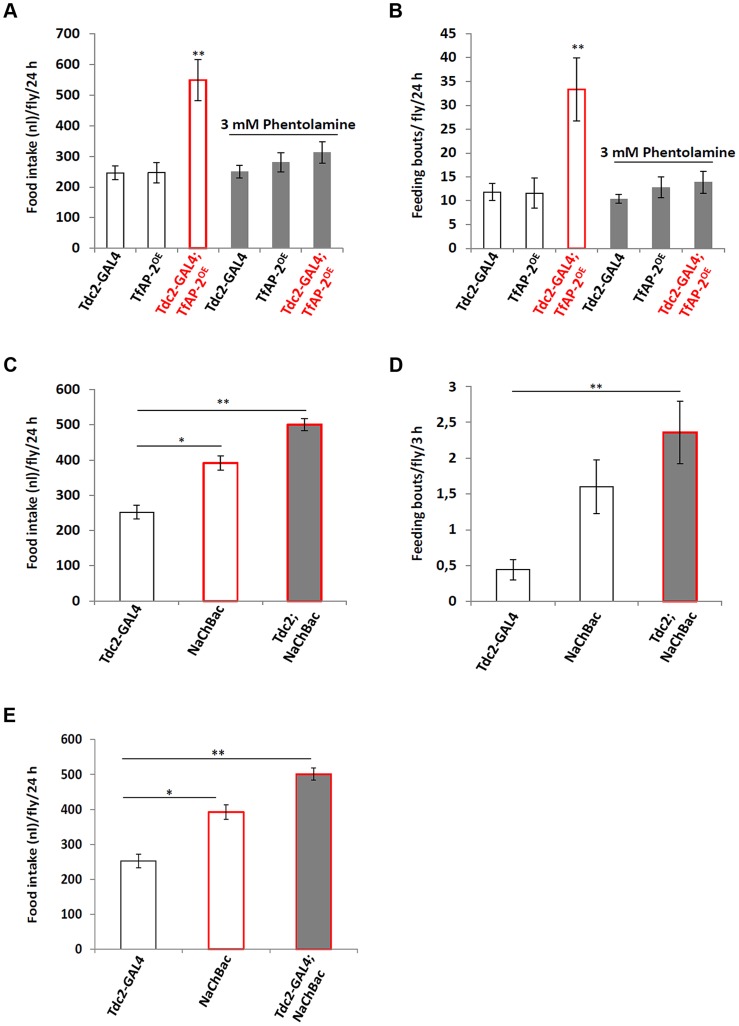
Octopamine regulates feeding behavior. (A,B) Male flies were fed 3 mM of the octopamine antagonist phentolamine before a CAFE assay was performed to assess total food intake (A) and the average number of feeding bouts (B) over a 24 h period in 5–7 days old adult males. Five males were used for each replicate and the assay was repeated at least 10 times for each genotype (A non-parametric Kruskal-Wallis ANOVA was performed with Dunn's post hoc test for multiple comparisons, P<0.005). (B,C) Overexpression of a bacterial sodium channel in octopaminergic neruons influences feeding behavior. A CAFE assay was used to assess total food intake over a 24 h period (C) and the average number of feeding bouts (D) over a 3 h period, during the first 3 h after lights-on, in 5–7 days old adult males. Five males were used for each replicate and the assay was repeated at least 10 times for each genotype (one-way ANOVA with a Dunn's post hoc test for multiple comparisons, * <0.05, ** P<0.005). Error bars represent the SE (SEM). (E) Relative level of *Dsk* expression in males where NaChBac was overexpressed in octopaminergic neurons. (n = 10 qPCR runs). In E a double asterisk indicates significant difference one-way ANOVA with a Dunn's post hoc test for multiple comparisons, * P<0.05, ** P<0.005).

To determine if *TfAP-2* and *Twz* were regulating feeding through octopamine signaling, we fed flies 3 mM of the octopamine antagonist phentolamine, a concentration known to reduce *TfAP-2^OE^* induced hyperactivity to control levels, while not reducing the activity of controls [6]. A CAFE assay was performed to determine the total food intake and the average number of feeding bouts per fly over 24 h. While *Tdc2-GAL4^+/−^* and *TfAP-2^OE+/−^* control flies ate 246.4 nl (SE ±22.2) and 246.8 nl (SE ±32.9) respectively, similar to before, overexpressing *TfAP-2* in octopaminergic neurons significantly increased the total food intake to 548.8 nl (SE ±66.3, P<0.005). Feeding 3 mM phentolamine had no significant affect on the total food intake of control flies, but significantly reduced the total intake of *TfAP-2^OE^* males to control levels (312.5 nl, SE ±34.7, P = 0.573) (Figure 4A). Overexpressing *TfAP-2* in octopaminergic neurons significantly increased the number of feeding bouts (33.3 fb, SE ±6.6, P<0.005), while *TfAP-2^OE^* flies fed 3 mM phentolamine only had 13.9 fb (SE ±2.3, P = 0.642 compared to *Tdc2-GAL4^+/−^* controls) (Figure 4B). Interestingly, feeding control flies 3 mM phentolamine had no significant affect on the number of feeding bouts. Finally, in order to determine if octopamine had a direct effect on consummatory behavior, we expressed a UAS-transgene for the voltage-activated, bacterial sodium channel NaChBac [16] in octopaminergic neurons using the *Tdc2-GAL4* driver and then performed a CAFE assay as before. Of notable interest, overexpressing NaChBac in octopaminergic neurons significantly increased total food intake over a 24 h period (501.1 nl, SE ±16.9, P<0.005) compared to the *Tdc2-GAL4^+/−^* control males (252.3 nl, SE ±18.9) (Figure 4C). We noticed that flies usually feed within the first 3 h after lights on, so we videoed the flies during this time and counted the number of feeding bouts. Overexpressing NaChBac in octopaminergic neurons also significantly increased the number of feeding bouts over a 3 h period (2.36 fb, SE ±0.43, P<0.05) compared to the *Tdc2-GAL4^+/−^* control males (0.44 feeding bouts, SE ±0.14) (Figure 4D). In the CAFE assay the UAS-NaChBac control line increased both total food intake and feeding bouts, indicating that this line has low levels of expression even without the GAL4 driver. These results indicate that TfAP-2 induced feeding behavior requires octopamine signaling. Furthermore, increasing octopamine signaling is sufficient to induce consummatory behavior in adult males.

Next to ascertain if octopamine signaling was directly influencing *Dsk* transcript levels we expressed the voltage-activated, bacterial sodium channel NaChBac [16] in octopaminergic neurons using the *Tdc2-GAL4* driver and performed qPCR to observe *Dsk* levels (Figure 4E). The *Dsk* transcript levels of *Tdc2-GAL4^+/−^* males was set as 100%, represented as 1 on the graph. Compared to *Tdc2-GAL4^+/−^* controls (SE ±0.06), males where NaChBac was overexpressed in octopaminergic neurons had significantly more *Dsk* transcript (1.75-fold, SE ±0.04, P<0.005) (Figure 4E). From this result we conclude that activating octopaminergic neurons is sufficient to induce *Dsk* expression in the CNS.

### Dsk inhibits feeding behavior

**Figure 5:**
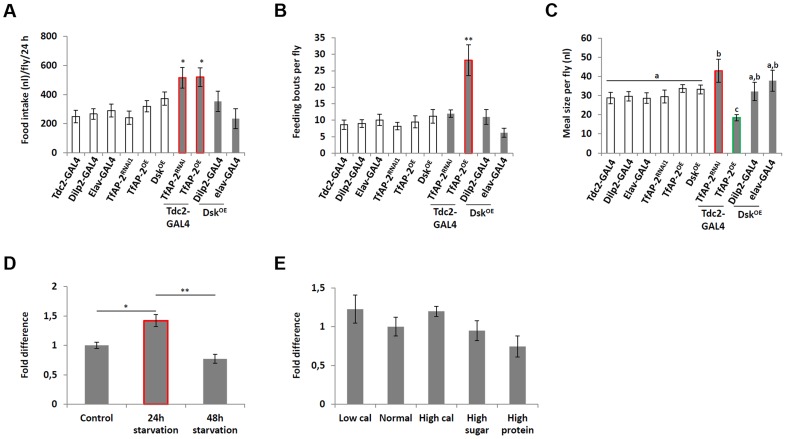
*Dsk* overexpression does not affect feeding behavior. (A–C) A CAFE assay was used to assess the total food intake (B), the average number of feeding bouts (C) and meal size (D) over a 24 hour period in 5–7 days old adult males. Five males were used for each replicate and the assay was repeated at least 10 times for each genotype. (D) Relative levels of *Dsk* transcript in the heads of flies starved either for 24 or 48 h. (E) Relative levels of *Dsk* transcript in the heads of flies kept on various diets for 5 days. (D,E) RNA was collected from the heads of 50, 5–7 day old, males for each genotype. qPCR was repeated at least 7 times for each transcript. (A,B and D: A single asterisk indicates significant difference, a non-parametric Kruskal-Wallis ANOVA was performed with Dunn's post hoc test for multiple comparisons with Dunn's post hoc test for multiple comparisons, P<0.05, while a double asterisk indicates significant difference P<0.005). (C: Different letters indicate similar groups (i.e. ‘a’ is significantly different than ‘b’ or ‘c’ and so on, A non-parametric Kruskal-Wallis ANOVA was performed with Dunn's post hoc test for multiple comparisons, P<0.05). Error bars represent the SE (SEM).

Overexpression of *Dsk* in the insulin-producing cells (IPCs) is sufficient to mimic *TfAP-2^OE^* induced male aggression phenotypes [6]. To discover if overexpression of *Dsk* could mimic *TfAP-2^OE^* feeding phenotypes we performed a CAFE assay on flies fed *ab lib*. *Dsk* was overexpressed in the IPCs (*Dilp2-GAL4*), as well as pan-neuronally (*elav-GAL4*), and the feeding behavior phenotype was compared to males where *TfAP-2* was either knocked down (*TfAP-2^RNAi^*) or overexpressed (*TfAP-2^OE^*) in octopaminergic neurons. Similar to what was observed before knocking down or overexpressing *TfAP-2* increased total food intake. However, overexpressing *Dsk* pan-neuronally or specifically in the IPCs had no affect on the amount of food adult males consumed (Figure 5A). Also, flies overexpressing *Dsk* in either the IPCs (11.2 fb, SE ±2.3, P = 0.93) or pan-neuronally (6.2 fb, SE ±1.4, P = 0.23) had no significant change in the number of feeding bouts, when compared to controls (8.7 fb, SE ±1.4) (Figure 5B). Unlike *TfAP-2^OE^* males, which had an average meal size of 18.4 nl (SE ±1.7, P<0.005), control flies ate on average 28.5 nl per meal (SE ±3.1) and males overexpressing *Dsk* in IPCs or pan-neuronally ate normal size meals, 32.1 nl (SE ±4.8, P = 0.56) and 37.8 nl (SE ±5.4, P = 0.16), respectively (Figure 5C).

Starvation had a significant effect on *TfAP-2* transcription and to a lesser degree on *Twz* expression (Figure 1A). To understand if starvation influenced *Dsk* transcriptional levels we performed qPCR analysis on adult males starved for 24 h or 48 h. Compared to flies fed *ab lib*, starving males for 24 h significantly increased *Dsk* expression (1.42-fold, SE ±0.10, P<0.05 compared to controls), yet after 48 h of starvation *Dsk* expression was back to control levels (Figure 5D). Finally, we determined if macronutrient content influenced *Dsk* expression, but none of the diets had a considerable affect, though a slight increase in Dsk expression was observed in flies fed a low calorie diet, and a decrease in expression was observed in flies fed a high proteins diet (Figure 5E). From these results is appears dietary conditions regulate *Dsk* transcription in a manner similar to *Vmat*.

### Octopamine and Dsk signal in a negative feedback-loop

**Figure 6:**
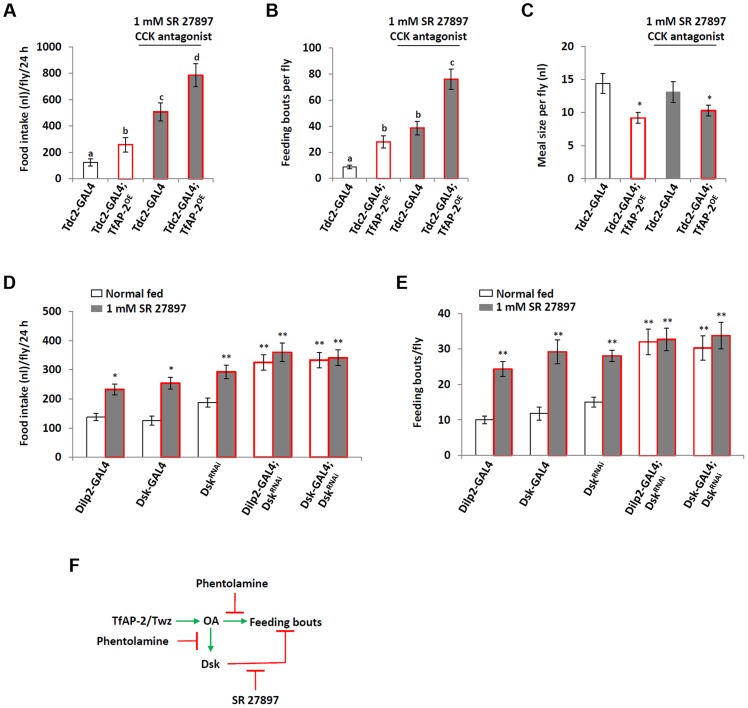
Dsk signals to inhibit feeding. (A–C) Male flies were fed 1 mM of the CCK antagonist SR 27897 before a CAFE assay was performed. (A) Feeding control and *TfAP-2^OE^* males SR 27897 increased total food intake, (B) and the number of feeding bouts, but (C) did not have an effect on the average meal size. Five males were used for each replicate and the assay was repeated at least 10 times (D,E) Knocking down *Dsk* in either the insulin-producing cells or all Dsk expressing cells inhibits the increase in total food intake (D) and feeding bouts (E) observed when flies are fed 1 mM of the CCK antagonist SR 27897. (F) Schematic drawing of octopamine and Dsk feedback lop to regulate the number of feeding bouts. Phentolamine is an octopamine antagonist, SR 27897 is a mammalian CCK antagonist. In A and B, different letters indicate similar groups (i.e. ‘a’ is significantly different than ‘b’ or ‘c’ and so on, A non-parametric Kruskal-Wallis ANOVA was performed with Dunn's post hoc test for multiple comparisons, P<0.05). In C a single asterisk indicates significant difference from the *Tdc2-GAL4* normal fed control (A non-parametric Kruskal-Wallis ANOVA was performed with Dunn's post hoc test for multiple comparisons, P<0.05). In D and E a double asterisk indicates significant difference from the *Dilp2-GAL4* normal fed control (A non-parametric Kruskal-Wallis ANOVA was performed with Dunn's post hoc test for multiple comparisons, P<0.005). Error bars represent the SE (SEM).

Overexpressing *Dsk* pan-neuronally (*elav-GAL4*) induced a slight, albeit not significant, decrease in the number of feeding bouts compared to controls (Figure 5B). This, along with the earlier study that determined loss of *Dsk* caused starved flies to overeat, could mean *Dsk* actually signals to inhibit feeding behavior [12]. In order to understand if *TfAP-2* was regulating feeding behavior through *Dsk* in a negative feedback loop, we fed control (*Tdc2-GAL4^+/−^*) and *TfAP-2^OE^* males the mammalian CCK antagonist SR 27897 before performing a CAFE assay [17], [18]. The CCK antagonist significantly increased total food intake in adult males (507.3 nl, SE ±68.2, P<0.005), compared to controls fed normal food (124.7 nl, SE ±55.4). Similar to what was observed previously, *TfAP-2^OE^* males ate significantly more food (258.3 nl, SE ±1.4, P<0.005) than controls (Figure 6A). Feeding these males the CCK antagonist had an additive effect, causing a drastic increase in total food intake (786.1 nl, SE ±88, P<0.005). The CCK antagonist also significantly increased the number of feeding bouts in *Tdc-GAL4^+/−^* controls (38.6 fb, SE ±5.2, P<0.005), which was more similar to *TfAP-2^OE^* males (28 fb, SE ±4.7, P<0.005 compared to controls) and significantly higher than controls (8.7 fb, SE ±1.4) (Figure 6B). Feeding the antagonist to *TfAP-2^OE^* males increased the number of feeding bouts even further to 76 (SE ±7.9, P<0.005). The antagonist SR 27897 had no effect on the meal size of either control or *TfAP-2^OE^* males (Figure 6C).

To verify *Dsk* function in feeding behavior, as well as determine if the mammalian CCK antagonist SR 27897 was actually inhibiting *Drosophila* Dsk signaling, we fed flies 1 mM SR 27897 where *Dsk* was knocked down in either the IPCs using the *Dilp2-GAL4* driver or in all *Dsk* expressing cells using the *Dsk-GAL4* driver [19], [20] and performed a CAFE assay. *Dilp2-GAL4, Dsk-GAL4* and *UAS-Dsk^RNAi^* heterozygous controls ate 137.5 nl (SE ±12.3), 125.5 nl (SE ±15.3) and 187.5 nl (SE ±15) respectively, feeding these controls 1 mM SR 27897 significantly increased their total food intake over a 24 h period (*Dilp2-GAL4*, 232.8 nl, SE±18.7, P<0.05; *Dsk-GAL4*, 254.2 nl, SE ±20.3, P<0.05; *UAS-Dsk^RNAi^*, 292.9 nl, SE ±23.4, P<0.05). Knocking down *Dsk* in the IPCs (*Dilp2-GAL4*) or in all *Dsk* expressing cells (*Dsk-GAL4*) significantly increased the total food intake over a 24 h period, but feeding these flies the CCK antagonist SR 27897 did not further increase total intake (Figure 6D). The heterozygous controls *Dilp2-GAL4, Dsk-GAL4* and *UAS-Dsk^RNAi^* had 10 (SE ±1.1), 11.8 (SE ±1.8) and 15 (SE ±1.4) feeding bouts over a 24 h period, respectively (Figure 6E). Feeding these controls 1 mM SR 27897 significantly increased the number of feeding bouts (*Dilp2-GAL4^+/−^*: 24.4 fb, SE ± 2.1, P<0.005; *Dsk-GAL4^+/−^*: 29.2 fb, SE ±3.4, P<0.005; *UAS-Dsk^RNAi+/−^*: 28.1, SE ±1.6, P<0.005). Knocking down *Dsk* in the IPCs or in all *Dsk* expressing cells significantly increased the number of feeding bouts (*Dilp2-GAL4; Dsk^RNAi^*: 32 fb, SE ±3.6, P<0.005; *Dsk-GAL4;Dsk^RNAi^*: 30.3 fb, SE ±3.4, P<0.005), but feeding these flies the CCK antagonist did not further increase feeding bouts (*Dilp2-GAL4;Dsk^RNAi^*: 32.7 fb, SE ±3.2, P = 0.885; *Dsk-GAL4;Dsk^RNAi^*: 33.8 fb, SE ±3.8, P<0.861) (Figure 6E). From the above data we present the following model: TfAP-2 and Twz regulate the production and secretion of octopamine, which in turn initiates feeding, while at the same time, in a negative feedback loop, octopamine induces the expression of *Dsk* to inhibit feeding frequency (Figure 6F).

### Co-localization of the neuronally expressed AP-2β and Kctd15 in the mouse brain

**Figure 7:**
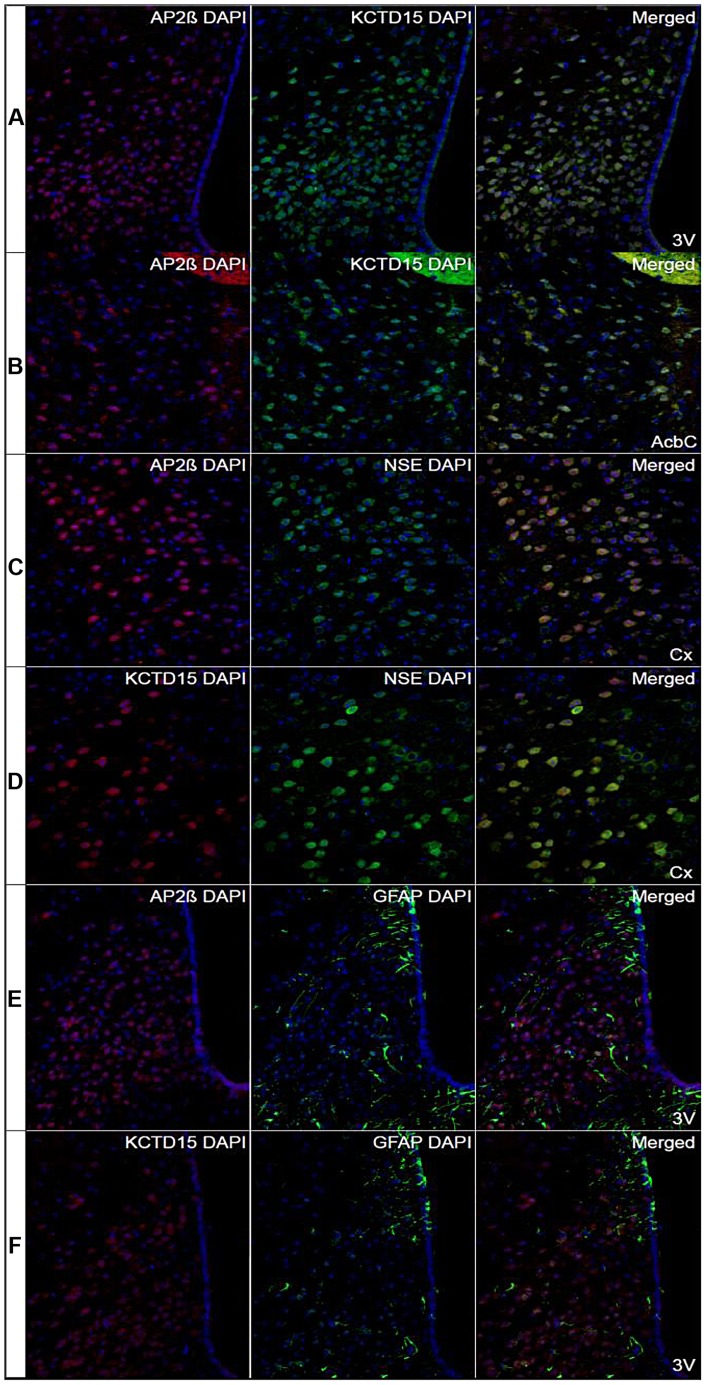
Co-localization of AP2β and KCTD15 immunoreactivity in hypothalamus and striatum. Immunohistochemistry performed on sections from mouse brain with the nucleus marker DAPI labeled in blue. (Row A) The first row show extensively overlapping expression of AP2β (red) and KCTD15 (green) in Arc and VMH in hypothalamus close to the third ventricle (3V) (Bregma −1.58). (Row B) AP2β immunoreactivity (red) was highly overlapped with the KCTD15 immunoreactivity (green) in AcbC in the ventral striatum (Bregma 1.10). (Row C) The neuronal marker NSE (green) and AP2β (red) co-localized in the cerebral cortex (Cx) (Bregma 1.10). (Row D) The protein expression of NSE (green) and KCTD15 (red) overlapped in the cerebral cortex (Bregma −1.46). (Row E) The immunoreactivity of the astrocyte marker GFAP (green) did not overlap with the expression of AP2β (red) in the hypothalamus, close to the third ventricle (3V) (Bregma −1.46). (Row F) The astrocyte marker GFAP (green) did not overlap with KCTD15 (red) in the hypothalamus (Bregma −1.34). Pictures were taken with 20× magnification.

To ascertain if, similar to *Drosophila*, the mammalian *Tfap2b* (encoding AP-2β) and *Kctd15* genes could be involved in regulating feeding behavior, immunohistochemistry was performed to investigate if AP-2β and Kctd15 co-localized in the mouse brain, and known markers for neurons and glial cells were used to investigate in what cell type they were expressed, see Figure 7. AP-2β and Kctd15 had similar expression patterns with high and exclusive expression in parts of cerebral cortex, cerebellum and hypothalamus. Overlapping expression was seen for AP-2β and Kctd15 in the arcuate hypothalamic nucleus (Arc) and the ventromedial hypothalamic nucleus (VMH) (Figure 7A). Co-localization of AP-2β and Kctd15 immunoreactivity was also seen in the core of the accumbens nucleus (AcbC) in ventral striatum (Figure 7B). These are all areas that are known to be involved in the regulation of food intake [21], [22]. The neuron-specific enolase (NSE) was used to visualize the neuronal expression of AP-2β and Kctd15. Interestingly, overlapping expression was seen for AP-2β and the neuronal marker NSE [23] in cerebral cortex (Figure 7C). Kctd15 showed a similar pattern with strong co-localization with NSE in cerebral cortex (Figure 7D). The expression of the astrocyte marker glial fibrillary acidic protein (GFAP) [24] did not overlap with neither AP-2β nor Kctd15 in the brain (Figure 7E and 7F, respectively). To conclude, AP-2β and Kctd15 immunoreactivity co-localize in specific areas of the mouse brain and are localized to neurons.

### Regulation of Tfap2b and Kctd15 by food intake in mouse hypothalamus

**Figure 8:**
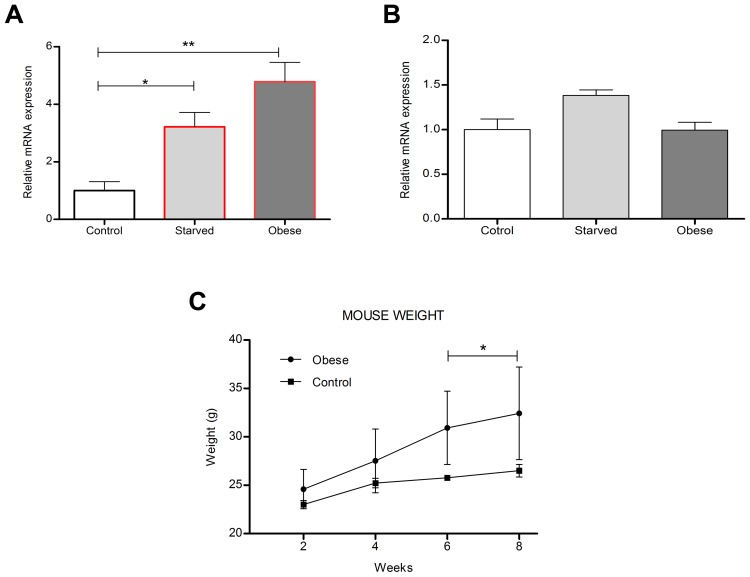
Diet regulates mouse hypothalamic *Tfap2b* and *Kctd15* transcript levels. (A,B) Relative level of (A) *Tfap2B* and (B) *Kctd15* expression in the hypothalamus from starved or obese male mice (n = 10 qPCR runs; one-way ANOVA with Bonferroni post hoc test for multiple comparisons, * P<0.05, ** P<0.005). (C) Mice were either fed a normal or high fat diet for 8 weeks. By week six mice raised on a high fat diet were significantly higher in weight than control mice (Two-way ANOVA with P<0.05 was calculated to ensure that the obese mice were significantly heavier than the controls). Error bars represent SD.

To study how the dietary status influenced the mRNA expression of Kctd15 and Tfap2b in the hypothalamus, mice were assigned to different food restrictions; 1) fed normal chow-, 2) fed normal chow, but food-deprived for 24 h- and 3) fed high fat diet to induce obesity before analyses. The expression levels of *Tfap2b* and *Kctd15* in normal chow fed mice were sat at 100%, represented as 1 in the graphs (Figure 8A and 8B). Both genes were up-regulated by fasting, but only *Tfap2b* was significantly changed, with relative expression at 3.22 (SD ±0.50, P>0.05) for *Tfap2b* and 1.38 (SD ±0.06) for *Kctd15* (Figure 8A). Further, *Tfap2b* was also affected by the high-fat diet with up-regulated relative expression levels at 4.78 (SD ±0.67, P>0.01), while *Kctd15* was unaffected by obesity (0.99±0.09 (±SD) (Figure 8B). Mice fed a high-fat diet for 6–8 weeks had significantly higher weight than mice fed normal chow (Figure 8C). Hence dietary status affects the expression of *Tfap2b* and to a much lesser extent *Kctd15*, in the hypothalamus in mice, this is similar to what was observed for the *Drosophila* homologues *TfAP-2* and *Twz* (Figure 1A and 1B)

### AP-2β and KCTD15 directly interact in hypothalamus cells

**Figure 9:**
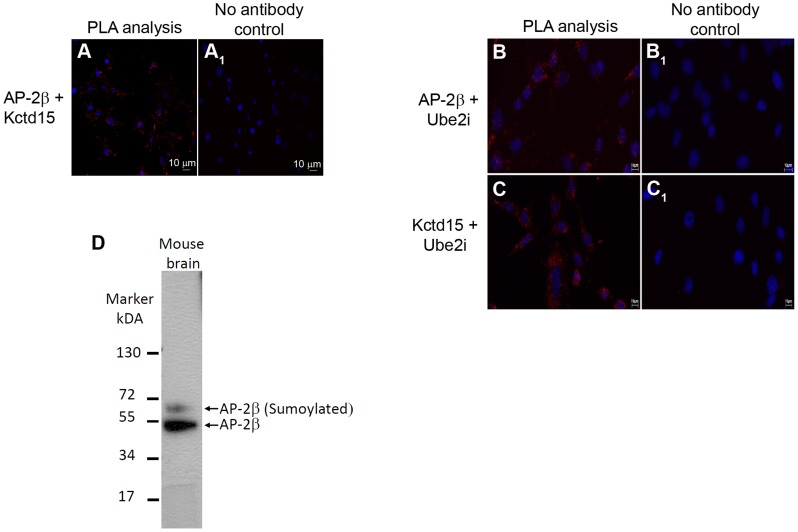
Detection of AP-2β and Kctd15 interactions in mHypoE-N25/2 cells using PLA. The images were acquired in single z-plane. PLA signals are shown in red (increased intensity projection) and the nuclei in blue (as stained by DAPI). Pictures were taken with 20× magnification. (A) PLA interaction between AP2β and Kctd15, (A_1_) negative control without primary antibodies. Detection of AP-2β, Kctd15 and Ube2i interactions in mHypoE-N25/2 cells using PLA. The images were acquired in single z-plane. PLA signals are shown in red (increased intensity projection) and the nuclei in blue (as stained by DAPI). Pictures were taken with 20× magnification. (B) PLA interaction between AP-2β and Ube2i, (B_1_) negative control without primary antibodies. (C) PLA interaction between Kctd15 and Ube2i, (C_1_) negative control without primary antibodies. (D) Western analysis of mouse proteins, recovered from the entire brain, was performed using an anti-AP-2β antibody. Two bands were visible on the gel, the lower band corresponded to the predicted size of AP-2β of 48 kDa, while the upper band corresponds to sumoylated AP-2β, which should be should be ∼60 kDa.

To ascertain if AP-2β and Kctd15 can directly interact *in vivo*, a proximity ligation assay (PLA) was performed using mHypoE-N25/2 cells. PLA can readily detect and localize proteins with single molecule resolution, allowing for the determination of directly interacting proteins [25]. Using this method, PLA signals were observed evenly distributed throughout the mHypoE-N25/2 cells, where both AP-2β and Kctd15 primary antibodies were added (Figure 9A, red dots), while no signals were observed in the control cells lacking primary antibodies (Figure 9A_1_). All cells were counter stained with DAPI to highlight the nuclei (blue staining).

In mice AP-2β interacts with the sumoylation enzyme Ube2i [26] and in *Drosophila* Twz interacts directly with the *Drosophila* Ube2i homologue Lesswright (Lwr) [9]. Furthermore, mouse AP2γ is sumoylated [26] and we have evidence that mouse AP-2β is also sumoylated (Figure 9D). Western blot analysis of mouse proteins recovered from the entire brain was performed using an anti-AP-2β antibody. Two bands were visible on the gel, the lower band corresponded to the predicted size of AP-2β (48 kDa), the upper band corresponded to sumoylated AP-2β (∼60 kDa) (Figure 9D). This result led us to perform a proximity ligation assay (PLA) using mHypoE-N25/2 cells to understand if Kctd15 and AP-2β directly interact with Ube2i. Using this method PLA signals were observed in punctate points throughout the mHypoE-N25/2 cells, when either AP-2β or Kctd15 primary antibodies were added together with Ube2i antibodies (Figure 9B and 9C respectively, red dots), while no signals were observed in cells lacking primary antibodies (Figure 9B_1_ and 9C_1_ respectively).

## Discussion

Previously we have shown that the obesity-linked homologues *TfAP-2* and *Twz* interact genetically to positively regulate TfAP-2 activity [6]. This interaction could be regulated by orexigenic signals, including starvation or low macronutrient meal content (see figures 1A–C). In order to increase octopamine signaling, *TfAP-2* overexpression induces the expression of genes involved in producing and secreting octopamine, for example *Tyramine β hydroxylase* (*Tbh*) and *Vesicular monoamine transporter* (*Vmat*) [6]. Octopamine signaling initiates consummatory behavior, and in a negative feedback loop to prevent overeating, signals to induce the expression of *Dsk*; the Dsk anorexigenic signal then inhibits feeding. Intriguingly, we demonstrate that loss of *TfAP-2* or overexpression of *TfAP-2* in octopaminergic neurons induces an overeating phenotype, but by different mechanisms. Knocking down *TfAP-2* caused flies to eat larger meals, but did not influence the number of meals per day; while ovexpressing *TfAP-2* significantly increased the number of meals taken per day. The overexpression phenotype can be explained by our model (see figure 6F). Increased octopamine signaling would induce flies to eat, but at the same time increase Dsk signaling to inhibit feeding. This feedback loop would lead to an increase number of feeding bouts, due to octopamine signaling, but premature cessation of food intake due to increased *Dsk* expression. The increased meal size due to loss of *TfAP-2* is harder to explain, but points to parallel pathways involved on regulating meal size. It was recently reported that similar to *Dsk*, another neuropeptide *Crustacean cardioactive peptide* (*Ccap*), *Drosophila* vasopressin/oxytocin homologue, is also expressed in the insulin-producing cells [27]. Intriguingly we have evidence that Ccap is involved regulating meal size in adult *Drosophila* (M. Williams, unpublished observations), both vasopressin and oxytocin have been shown to regulate meal size in mammals [28]–[30]

A recent paper demonstrated that the *Drosophila* noradrenalin analogue octopamine was involved in regulating consummatory behavior in larvae [13]. In that particular study it was determined that octopamine drives the response for continual eating of palatable food, unlike the NPY-like system that was shown to initiate eating of less palatable food under adverse conditions [31], [32]. Of note, chronic infusion of the mammalian octopamine analogue noradrenalin into the ventromedial hypothalamic nucleus (VMH) of rats induces obesity, most likely due to hyperphagia and increased levels of circulating insulin and triglycerides [33], [34]. Furthermore, dietary amino acid deficiency inhibited noradrenalin release within the VMH, probably stimulated by an aversion response to inhibit consummatory behavior [35]. Interestingly, we found that in the *Drosophila* CNS or mouse hypothalamus starvation and low macronutrient meal content induced the expression of *TfAP-2* (see figure 1A and 1B) or *Tfap2b* (see figure 8A and 8B). Furthermore, when we stained mouse brains to look for possible *Tfap2b* (encoding AP-2β) and *Kctd15* interactions, we observed a strong co-localization of AP-2β and Kctd15 within neurons located in the VMH. Moreover, overexpressing *TfAP-2* in *Drosophila* octopaminergic neurons induces hyperphagia, which could be rescued by an octopamine antagonist. Finally, hyperactivating octopaminergic neurons is sufficient to induce hyperphagia in adult male *Drosophila*. These findings suggest that in flies and mammals the initiation and cessation of consummatory behavior is controlled by a conserved signaling system.

CCK, a mammalian gastrointestinal hormone secreted by the gut when nutrients enter the lumen, binds to the cholecystokinin A receptor (CCKAR) located on vagus sensory terminals. The vagus nerve then delivers satiation signals to the nucleus of the solitary tract (NTS) [10], [11]. Under some experimental conditions exogenous CCK signaling elicits satiety and reduces meal size in several species [36]–[41]. Interestingly, our experiments show that although feeding wild-type flies a CCK antagonist has no affect on meal size, there is a significant increase in the number of feeding bouts (see figure 6B). Moreover, feeding *TfAP-2* overexpressing males, which already undergo significantly more feeding bouts than controls, a CCK antagonist had an additive effect. Finally, knocking down the *Drosophila* CCK homologue in the insulin-producing cells (IPCs) was sufficient to induce hyperphagia (see figure 6D and 6E). This differs somewhat to what is observed in mammals, where inhibition of CCK increases meal size. Although there have been no studies reporting the effect of noradrenalin on CCK transcription or signaling, it has been shown that CCK signaling to the VMH can inhibit noradrenalin release [42]. It could be that CCK released from the gut inhibits meal size, while CCK functions within the hypothalamus to block noradrenalin release, thus inhibiting the number of feeding bouts.

Previously, we reported that overexpression of *TfAP-2* or feeding *Drosophila* the octopamine analogue chlordimeform is sufficient to induce *Dsk* transcription, and that *Dsk* induction by *TfAP-2* overexpression could be blocked by feeding these flies an octopamine antagonist [6]. Here we demonstrate that specifically activating octopaminergic neurons using a bacterial sodium channel is sufficient to induce hyperphagia (see figure 4C and 4D). Also, a recent study demonstrated that octopamine signaling induced fervent feeding behavior when larvae were presented with palatable food [13]. Furthermore, it was shown that Dsk is necessary to inhibit well-fed larvae or adults from eating less palatable foods [12]. In both flies and mammals it is possible that there exists a conserved negative feedback loop to inhibit overeating, as well as allow for the differentiation between palatable or non-palatable foods.

Our results indicate that there is an epistatic interaction between *TfAP-2* and *Twz*. In all the assays performed, where *TfAP-2* was overexpressed, loss of *Twz* was able to rescue the *TfAP-2* overexpression phenotypes. Also, we have previously reported, and again shown here that *Twz* is necessary for proper *TfAP-2* transcription [6]. Furthermore, in mHypoE-N25/2 cells, which are derived from the mouse hypothalamus, a proximity ligation assay showed that throughout the cytoplasm mouse AP-2β and Kctd15 interact directly (see figure 9A). The question is what is the function of this interaction? In a yeast two-hybrid screen, using a human brain cDNA library, KCTD1 was identified as a binding partner for AP-2α, a paralogous protein to AP-2β. Transient transfection assays, using AP-2-binding site containing promoters, established that KCTD1 actively repressed AP-2α-mediated transactivation, demonstrating that the function of KCTD1 was to inhibit TFAP2α activity. This is not what we observed for *Drosophila TfAP-2* and *Twz*, where Twz was required for TfAP-2 activity. Mouse AP-2β interacts with Ube2i [26], a sumoylation enzyme, and in *Drosophila* Twz interacted directly with the *Drosophila* Ube2i homologue Lesswright (Lwr) in a large-scale yeast two-hybrid screen [9]. We show that in mHypoE-N25/2 cells mouse AP-2β and Kctd15, both directly interact with the sumoylation enzyme Ube2i. It is possible that Twz/Kctd15 acts like a scaffold where TfAP-2/AP-2β is either sumoylated or ubiquitinated, as many KCTD family members also interact with the ubiquitination apparatus [43], [44]. This post-translational modification might be required for TfAP-2/AP-2β activation. In both *Drosophila* CNS and mouse hypothalamus *TfAP*-2 and *Tfap2b* transcription levels went up after starvation. In *Drosophila* this increase in transcription required *Twz*. It could be that satiation levels regulate TfAP-2/Tfap2b and Twz/Kctd15 interactions, which in turn regulates TfAP-2/Tfap2b post-translational modification to increase TfAP-2/Tfap2b activity. During starvation or in conditions of dietary restriction TfAP-2 and Twz could interact to activate TfAP-2 and thus induce TfAP-2 expression (see figure 1A and 1C). Furthermore under extreme conditions, more Twz may be needed, this is supported by the fact that *Twz* was transcriptionally induced only after 48 h of starvation. We have demonstrated previously that *Twz* expression does not require TfAP-2 [6]. Although we have not demonstrated it, in the mouse hypothalamus under low nutritive conditions Tfap2b and Kctd15 could interact to initiate sumoylation and activation of Tfap2b, which could in turn induce consummatory behavior.

In conclusion, our data suggests that the human obesity-linked genes TFAP2B and KCTD15 could directly interact in regions of the brain known to regulate feeding behavior. We demonstrated that not only do the *Drosophila* homologues genetically interact, but in a mouse hypothalamic cell line the mouse homologues physically interact. Furthermore, AP-2β and Kctd15 co-localize in regions of the mouse hypothalamus known to regulate feeding behavior. In this model Kctd15 could act like a scaffold where AP-2β would bind to be either sumoylated, in a fashion similar to the Kctd1 and AP-2α, or possibly ubiquitinated. This post-translational modification would then change AP-2β function and allow it to induce noradrenalin signaling to induce consummatory behavior.

## Materials and Methods

### Fly stocks and maintenance

w*, *P{w[+mW.hs] = GawB}elav[C155]*, w*; P{w[+mC] = Tdc2-GAL4.C}2, y^1^ w[*]; P{w[+mC] = UAS-AP-2.PB}a4-2, y^1^ w*; P{w[+mC] = UAS-NaChBac-EGFP}4 and the RNAi lines *y1 v1; P{TRiP.JF01908}attP2 Dsk*, *y1 v1; P{TRiP.JF03500}attP2 Tfap-2* (referred to as *TfAP2^RNAi2^*) and *y1 v1; P{TRiP.JF01867}attP2 Twz* (referred to as *Twz^RNAi2^*) were received from the Bloomington Stock Center (Table 1). *TfAP-2 (y^1^w^3^; P{KK109052}VIE-260B*, referred to as *TfAP-2^RNAi1^*) and *Twz (y^1^w^3^*; *P{KK107922}VIE-260B*, referred to as *Twz^RNAi1^*) RNAi flies were obtained from the Vienna Drosophila RNAi Centre (VDRC, Vienna, Austria) (Table 1). *w; Dilp2-GAL4* was a gift from Dr. Eric Rulifson [45]; *w; Dsk-GAL4* and *w*; *UAS-Dsk* line were a gift from Dr. Barry Ganetzky [19], [20]. All flies, unless otherwise stated, were maintained on enriched Jazz mix standard fly food (Fisher Scientific). Flies were maintained at 25°C in an incubator at 60% humidity on a 12∶12 light∶dark cycle. Flies crossed to GAL4 drivers and controls were raised at 18°C until the adults emerged; once collected adults were raised at 29°C for the appropriate times. In all assays, the GAL4 drivers and UAS transgenic flies were crossed to *w^1118^* flies and their F1 progeny used as controls.

**Table 1:** Information about RNAi lines, including possible number of off-target genes.

Trans- Formant ID	Construct ID	Library	CG number	Gene	On targets	Off targets	s19	CAN repeats
101552	109052	KK	CG7807	TfAP-2	1	0	1	2
110265	107922	KK	CG10440	Tiwaz	1	3	0.99	3
P{TRIP.J03500}	JF03500	TRIP-1	CG7807	TfAP-2	1	0	1	2
P{TRIP.JF01852}	JF01852	TRIP-1	CG10440	Tiwaz	1	0	1	2

### Capillary feeding (CAFE) and antagonist assay

This method was modified from Ja et al. 2007 [15]. A vial, 9 cm by 2 cm (height×diameter), containing 1% agarose (5 cm high) to provide moisture and humidity for the flies, was used for this assay. A calibrated capillary glass tube (5 µl, VWR International) was filled with liquid food which contains 5% sucrose, 5% yeast extract and 0.5% food-coloring dye. Mineral oil was used to prevent the liquid food from evaporating. The vial was covered with paraffin; a capillary tube was inserted from the top through the paraffin. The experimental set up was kept at 25°C and activity was recorded for 24 h using a HD camera (Panasonic SDS90). The initial and final food level in the capillary tube was marked to determine total food intake. Number of feeding bouts per fly was counted from the recording; average meal size was calculated by dividing the total food intake by the number of feeding bouts. Five 5–7 day old males per vial were used for this assay.

### Feeding after starvation

Twenty 5–7 day old males were maintained in a vial containing 1% agarose for 16 h. They were then transferred to normal food vials (5% sucrose, 5% yeast extract and 1% agarose) and allowed to feed for 20 minutes, then transferred to a second food vial containing normal food (5% sucrose, 5% yeast extract and 1% agarose) and 2% blue or green food-coloring dye (Dr. Oetker) and allowed to feed for 15 minutes. After this time the abdomen of each fly was observed using dissecting microscope (DV4, Zeiss) and the color of the gut was scored. The percentage of overeaters was calculated by dividing those with blue colored guts by the total number of flies observed.

### Antagonist assays

Newly eclosed *TfAP-2* overexpressing male flies were collected and isolated on normal food for 3 days. After this time they were fed by CAFE assay method [15]. Calibrated capillary glass tubes (5 µl, VWR) were filled with either 1 mM SR 27897 [17], [18] or 3 mM of Phentolamine [46], prepared in liquid food (5% of sucrose and 5% yeast extract), a layer of mineral oil was used to prevent the liquid food evaporation from the capillary tube. These tubes were inserted from the top through paraffin film into the chambers. After 2–3 days of feeding the flies were used for the various assays performed.

### RNA purification

The phenol-chloroform method was used for RNA extraction from tissue samples [47]. Fifty fly heads were homogenized with 800 µl TRIzol (Invitrogen, USA), 200 µl Chloroform (Sigma-Aldrich) was added and samples were centrifuged at 12000 rpm for 15 minutes at 4°C. The aqueous layer, which contained RNA, was separated and 500 µl isopropanol (Solvaco AB, Sweden) was added.The RNA was precipitated by storing the samples at −32°C for 2 h. Samples were centrifuged at 12000 rpm for 10 minutes at 4°C, to collect the RNA pellets, which were then washed with 75% ethanol (Solvaco AB, Sweden) to remove the organic impurities. Samples were allowed to air dry to remove any traces of ethanol. Dried RNA pellets were dissolved in 21.4 µl of RNAse free water (Qiagen GmBH, Germany) and 2.6 µl of DNAse incubation buffer (Roche GmBH, Germany). The samples were incubated at 75°C for 15 minutes to ensure complete dissolution of RNA-pellets. 2 µl of DNAse I (10 U/µl, Roche GmBH, Germany) was added to each sample, and incubated at 37°C for 3 hr to remove DNA contamination. DNAse was deactivated by incubating the samples at 75°C for 15 minutes. Removal of DNA was confirmed by PCR using Taq polymerase (5 U/µl, Biotools B & M Labs, Spain), followed by agarose gel electrophoresis. The RNA concentration was measured using a nanodrop ND 1000 spectrophotometer (Saveen Werner).

### cDNA synthesis

cDNA was synthesized from RNA template using dNTP 20 mM (Fermentas Life Science), random hexamer primers and M-MLV Reverse Transcriptase (200 U/µl, Invitrogen, USA) by following manufactures instructions. cDNA synthesis was confirmed by PCR followed by agarose gel electrophoresis.

### qRT-PCR

Relative expression levels of three housekeeping genes (*EF-1, Rp49* & *RpL11*) and of the genes of interest were determined with quantitative RT-PCR (qPCR). Each reaction, with a total volume of 20 µl, contained 20 mM Tris/HCl pH 9.0, 50 mM KCl, 4 mM MgCl_2_, 0.2 mM dNTP, DMSO (1∶20) and SYBR Green (1∶50000). Template concentration was 5 ng/µl and the concentration of each primer was 2 pmol/µl. Primers were designed with Beacon Designer (Premier Biosoft) using the SYBR Green settings. All qPCR experiments were performed in duplicates; for each primer pair a negative control with water and a positive control with 5 ng/µl of genomic DNA were included on each plate. Amplifications were performed with 0.02 µg/ml Taq DNA polymerase (Biotools, Sweden) under the following conditions: initial denaturation at 95°C for 3 min, 50 cycles of denaturing at 95°C for 15 sec, annealing at 52.8–60.1°C for 15 sec and extension at 72°C for 30 sec. Analysis of qPCR data was performed using MyIQ 1.0 software (Bio-Rad) as previously reported [48]. Primer efficiencies were calculated using LinRegPCR [49] and samples were corrected for differences in primer efficiencies. The GeNorm protocol described by Vandesompele et al. [50] was used to calculate normalization factors from the expression levels of the housekeeping genes. Grubbs' test was performed to remove outliers. Differences in gene expression between groups were analyzed with ANOVA followed by Fisher's PLSD test where appropriate. P<0.05 was used as the criterion of statistical significance. The following primers were used: *EF-1* F: 5′-GCGTGGGTTTGTGATCAGTT-3′, R: 5′-GATCTTCTCCTTGCCCATCC-3′; *Rp49* F: 5′-CACACCAAATCTTACAAAATGTGTGA-3′, R: 5′-AATCCGGCCTTGCACATG-3′; *RpL11* F: 5′-CCATCGGTATCTATGGTCTGGA-3′, R: 5′-CATCGTATTTCTGCTGGAACCA-3′, *TfAP-2* F: 5′-CTAAGAGCAAGAACGGAG-3′, R: 5′-AACCAAGGATGTCAGTAG-3′; *Tiwaz* F: 5′-GCCACATTCTGAACTTTATG-3′, R: 5′-CACCAAATAGTTGCCATT-3′; *Dsk* F: 5′-CCGATCCCAGCGCAGACGAC-3′, R: 5′-TGGCACTCTGCGACCGAAGC-3′

### Mouse experimental procedures

#### Ethical statement

All animal procedures were approved by the local ethical committee in Uppsala and followed the guidelines of European Communities Council Directive (86/609/EEC).

*Tissue collection and sectioning* - Adult, male C57BL/6J mice (Taconic M&B, Denmark) were anesthetized with an intraperitoneally injection of sodium pentobarbital (90 mg/kg IP; Apoteksbolaget, Sweden). Transcardial perfusion was performed through the left ventricle with phosphate-buffered saline (PBS), followed by 4% formaldehyde (HistoLab, Sweden), and brains were excised. Tissues were stored in 4% formaldehyde over night and sections were made by fixation in zinc-formalin (Richard-Allan Scientific, USA) for 18–24 h at 40°C, before dehydration and paraffin infusion with a Tissue-Tek vacuum infiltration processor (Miles Scientific, USA). Sections were cut (7 µm) using a Microm 355S STS cool cut microtome, mounted on Superfrost Plus slides (Menzel-Gläser, Germany), dried over night at 37°C and stored at 4°C.

#### Fresh tissue collection

17 adult male C57BL/6J mice (Taconic M&B, Denmark) were used for fresh dissection of the hypothalamus. The animals were maintained in a temperature-controlled room on a 12-h light-dark cycle where they had free access to food and water at all times unless anything else stated. Six mice were fed normal chow until dissection; additional six mice were given chow, but were food deprived for 24 h before dissection to study starvation effects on gene expression. For diet-induced obesity, five mice were fed high fat western diet (R638, Lantmännen, Sweden) during eight weeks prior dissection. The diet-induced obesity mice weight was monitored weakly and compared with normal chow fed mice. Two-way ANOVA with P<0.05 was calculated to ensure that the obese mice were significantly heavier than the controls at the day of dissection. Mice were sacrificed by cervical dislocation during the light period and all dissections were performed on ice. A coronal brain matrix (Alto, 1 mm) was used to facilitate the excised of the hypothalamus. Tissues were placed in RNA-later (Invitrogen) for 2 h at RT before frozen at −80°C.

#### RNA extraction

Tissue collected from mice on the same diet was pooled before RNA extraction. RNA was extracted using Absolutely RNA Miniprep Kit (Agilent Technologies, USA). Briefly, the tissue was mixed with Lysis Buffer, β-mercaptoethanol and 1 mm RNAse free glass beads before homogenized using a Bullet blender (Averill Park, USA). The homogenate was spun through a prefilter spin cup in a Heraesus Fresco 21 centrifuge at maximum speed at RT, before mixed with 70% ethanol (Solveco, Sweden) in a 1∶1 ratio to precipitate the RNA. The solution was centrifuged through a RNA binding spin cup, followed by salt buffer washes. DNase Digestion buffer and RNase-Free DNase 1 were then added to the RNA binding spin cup and allowed to incubate for 15 min at 37°C followed by additional washes in salt buffer prior to RNA elution. Concentration was measured using a nanodrop ND-1000 spectrophotometer.

#### cDNA synthesis

Extracted RNA was used as template for cDNA synthesis. 2 µg RNA was added to a 2×RT buffer and 20×RT enzyme master mix (High Capacity RNA-to-cDNA Kit, Applied Biosystem, USA), final volume was adjusted to 20 µL with DEPC-treated water. Samples were incubated for 37°C for 60 min followed by 95°C for 5 min. The cDNA was diluted to 5 ng/µl template in sterile water.

#### Primer design and qPCR

All primers were designed using Beacon primer design 8 (Premier Biosoft). For sample amplification following primers were used *KCTD15* F: 5′-CACCAAGTACCCTGACTC-3′, R 5′-AATAATGTTGCTTGAGACTGT-3′ and *Tfap2b*: F 5′-TTACAGTCCTATACTCTCC-3′, R 5′-CTACGCTTCAGTCTTTAG-3′. Three different reference genes were run: *GADPH*: F 5′-GCCTTCCGTGTTCCTACC-3′, R 5′-GCCTGCTTCACCACCTTC-3′, *mRPL19*: F 5′-AATCGCCAATGCCAACTC-3′, R 5′-GGAATGGACAGTCACAGG-3′ and *histone H3b:* F 5′-CCTTGTGGGTCTGTTTGA-3′, R 5′-CAGTTGGATGTCCTTGGG-3′.

For each qPCR reaction iQ SYBR Green supermix (Bio-rad, Sweden) was used, to which 5 µl cDNA (5 ng/µl template) and 100 pmol/µl of each primer was added. Final volume was adjusted to 20 µL with water. Each sample was run in triplicate, a negative control for each primer pair and a positive control was included on each plate. All experiments were repeated twice. iCycler real-time detection instrument (Bio-Rad Laboratories) were used and the reaction followed these conditions: initial denaturation for 30 sec at 94°C followed by 45 cycles of 10 sec at 94°C, 30 sec at 53–63°C (optimal temperature for each primer pair) and 30 sec at 72°C. Thereafter a melting curve was performed by 81 cycles of 10 sec intervals where the temperature increased 0.5°C per cycle, starting from at 55°C.

Analyses were performed as previously described under the qRT-PCR section. MyIQ 1.0 software was used to analyse the qPCR data and primer efficiency was calculated using LinRegPCR. Grubbs test was performed to remove outliers in the primer efficiency calculations before correcting the samples for primers efficiency. A normalization factor was obtained by using the expression levels of the housekeeping genes for calculations in GeNorm. The normalization factor was then used to calculate the relative mRNA expression in the samples. Differences in gene expression between the diets were analysed with one-way ANOVA, where Bonferroni's multiple comparison test was used for post-hoc analysis. P>0.05 was used as significance.

#### Fluorescent immunohistochemistry on paraffin embedded sections

Sections were deparaffinized in X-trasolv (Medite histotechnik, Germany), rehydrated through a series of ethanol solutions ending up in water, followed by PBS washes. Antigen retrieval was performed by heating the sections to 100°C in 0.01 M citric acid pH 6.0 (Sigma-Aldrich, USA). After PBS washes, sections were incubated with 1° antibodies overnight at 4°C. After PBS rinsing, sections were incubated for two h in 2° diluted in supermix. Sections were washed in PBS, stained with DAPI (1∶1250, Sigma-Aldrich, USA) and mounted in DTG media with antifade (diazabicyclo(2.2.2)octane in glycerol). Pictures were taken using a fluorescent microscope (Zeiss Axioplan2 imaging) connected to a camera (AxioCam HRm) with the Carl Zeiss AxioVision version 4.7 software.

### Antibodies for immunohistochemistry

#### Primary antibodies

Rabbit-anti-AP2β (1∶100, Abcam, England), mouse-anti-KCTD15 (1∶100, Abcam, England), chicken-anti-NSE (1∶200, Abcam, England) or chicken-α-GFAP (1∶800, Abcam, England) antibodies diluted in supermix (Tris-buffered saline, 0.25% gelatin, 0.5% Triton X-100).

#### Secondary antibodies

Goat-anti-mouse-594, goat-anti-chicken-488, goat-anti-rabbit-594 or goat-anti-mouse-488 antibodies (1∶200, Invitrogen, USA).

### Proximity ligation assay (PLA)

The immortalized embryonic mouse hypothalamus cell line N 25/2 were grown (Detailed methods on cell culture maintenance can be found in SI Experimental procedures) on glass slides (coated with 10 µg/ml poly-L-lycine) were fixed in 4% paraformaldehyde (Sigma-Aldrich, USA) for 15 min and rinsed with PBS. The Duolink II fluorescence kit (orange detection reagents, Olink Biosciences, Sweden) was used to run *in situ* proximity assay (PLA) on the fixed cells according to manufacturer's instruction. Primary rabbit-anti-AP2β, mouse-anti-KCTD15, goat-anti-UBC9 antibodies (Abcam, England) were diluted 1∶100, 1∶100, 1∶200 correspondently in Antibody Diluent supplied by the kit. Negative controls were run without primary antibodies. Protein interactions were detected with PLA probes in combination anti-rabbit PLUS and anti-mouse MINUS, anti-rabbit PLUS and anti-goat MINUS or anti-mouse PLUS and anti-goat MINUS. Slides were mounted using Duolink *in situ* Mounting Medium with DAPI. Pictures were taken using a fluorescent microscope (Zeiss Axioplan2) connected to a camera (AxioCam HRm) with the Carl Zeiss AxioVision version 4.7 software (Carl Zeiss, Germany).

### Cell culture

The immortalized embryonic mouse hypothalamus cell line N 25/2 (mHypoE-N25/2, Cellutions Biosystems Inc., Canada) was cultured in DMEM cell culture medium (Invitrogen) with 10% Fetal Bovine Serum (Invitrogen), 1% Penicillin Streptomycin (Invitrogen), 1× Amphotericin B (Invitrogen) in a humidified atmosphere with 5% CO_2_ in air at 37°C. Growth medium was changed every 2 days and cells were split with trypsin regularly with a ratio of 1∶2–1∶3 usually every 3 days.

### Western analysis

We performed a western blot analysis of AP-2β in brain tissue from adult, male C57Bl6/J mice (Taconic M&B, Denmark). Briefly, the tissue was homogenized in homogenization buffer (50 mM Tris, 150 mM NaCl, 4 mM MgCl, 0.5 mM EDTA, 2% Triton X-100 and 1 mM Protease inhibitor PMSF (Sigma-Aldrich, USA) diluted in isopropanol). Protein concentrations were determined by protein assay DC (Bio-Rad, Hercules, USA) according to the manufacturer's instructions. Equal amounts of protein (200 µg or 13 µg/µl) were separated, together with PageRuler prestained protein ladder (Fermentas, Canada), on a Mini-Protean TGX gel (4–10%, Bio-Rad, Hercules, USA) in running buffer (0.1% SDS, 0.025 Tris base and 0.192 M glycine) by gel electrophoresis. The proteins were transferred to a Immobilon-P polyvinylidene fluoride (PVDF) membrane (Millipore, Billerica, USA) in transfer buffer (0.025 Tris base, 0.192 M glycine and 20% methanol) and pre-blocked for 1 h in blocking buffer (5% non-fat dry milk (Bio-RAD, Hercules, USA) diluted in 1.5 M NaCl, 0.1 M Tris, 0.05% Tween-20, pH 8.0). The membrane was cut in the middle, giving two membranes with equally loaded protein samples. One half of the membrane was hybridized with the primary antibody against AP-2β (diluted 1∶200, rabbit-anti-AP2β, Abcam, England). The other half of the membrane was hybridized with AP-2β primary antibody that was pre-blocked with excess of the same synthetic peptide (sequence (NH2-) MHSPPRDQAA IMLWKLVENV KYEDIYEDRH DGVPSHSSRL SQLGSVSQGP (-CONH2), Abcam, England) that was used to generate the antibody. The hybridization was then performed overnight at 4°C. After washes in water, the membranes were incubated for 1 h with horseradish peroxidase conjugated secondary antibody (diluted 1∶10000, goat-anti-rabbit, Invitrogen, USA) followed by detection with the enhanced chemiluminescent (ECL) method. The membranes were incubated for 3 min in a 1∶1 mixture of luminol/enhancer and peroxidase buffer solutions (Immun-Star HRP, Bio-Rad, Hercules, USA) and developed on High performance chemiluminescence film (GE healthcare, Waukesha, USA).

### Statistical analysis

Mean and standard error from all replicates of each experiment was calculated. All analysis was performed with GraphPad Prism 4, and used ANOVA with appropriate post hoc analysis for multiple comparisons. The type of analysis performed for each assay is specified in the appropriate Figure legend.

## Supporting Information

Figure S1 (A–B) Relative level of *TfAP-2* and *Twz* expression in octopaminergic neurons in males kept at either (A) 29°C or (B) 18°C, to verify the efficiency of the various UAS constructs. (C) Relative level of *Dsk* expression in Dsk expressing neurons in males, to verify the efficiency of the *UAS-Dsk^RNAi^* construct. (In all assays n = 10 qPCR runs). In A different letters indicate similar groups (i.e. ‘a’ is significantly different than ‘b’ or ‘c’ and so on, one-way ANOVA with Bonferroni post hoc test for multiple comparisons, P<0.05). In G a double asterisk indicates significant difference one-way ANOVA with Bonferroni post hoc test for multiple comparisons, ** P<0.005). Error bars represent the SE (SEM).
(TIF)

Text S1 Description of RNAi line verification: To verify the efficiency of the RNAi lines *y1 v1; P{TRiP.JF03500}attP2 Tfap-2* (referred to as *TfAP2^RNAi2^*) and *y1 v1; P{TRiP.JF01867}attP2 Twz* (referred to as *Twz^RNAi2^*) we crossed them to the *Tdc2-GAL4* driver and performed qPCR. To verify the efficiency of the *Dsk* RNAi line *y1 v1; P{TRiP.JF01908}attP2 Dsk,* we crossed it to *Dsk-GAL4*.
(DOCX)
