# Supplementary material for: Obesity-Linked Homologues TfAP-2 and Twz Establish Meal Frequency in Drosophila melanogaster
Source: PLoS Genet. 2014 Sep 4;10(9):e1004499. doi: 10.1371/journal.pgen.1004499 (PMC4154645; doi:10.1371/journal.pgen.1004499)
Supplement: Text S1 — Description of RNAi line verification: To verify the efficiency of the RNAi lines y1 v1; P{TRiP.JF03500}attP2 Tfap-2 (referred to as TfAP2RNAi2) and y1 v1; P{TRiP.JF01867}attP2 Twz (referred to as TwzRNAi2) we crossed them to the Tdc2-GAL4 driver and performed qPCR. To verify the efficiency of the Dsk RNAi line y1 v1; P{TRiP.JF01908}attP2 Dsk, we crossed it to Dsk-GAL4. (DOCX) [file pgen.1004499.s002.docx]

***New TfAP-2 and Twz RNAi lines functional***

Previously, we have published that the RNAi lines *Tfap2^RNAi1^*and *Twz^RNAi1^* are functional {Williams, 2014 #3954}, but in this project we added one additional RNAi line for each gene (*Tfap2^RNAi2^*and *Twz^RNAi2^*). Before beginning any assays, to clarify that the new RNAi lines were functioning properly we crossed them to the *Tdc2-GAL4* driver and performed quantitative RT-PCR (qPCR) to measure the level of *TfAP-2* and *Twz* transcript (Figure S1A). Flies were raised at 18^o^C until they eclosed, at which point to get maximal expression from the GAL4/UAS system they were collected and kept at 29^o^C for 5-7 days. Since raising flies at 18^o^C inhibits, but may not completely block GAL4 activity, to make sure the effects we observed were not due to a developmental phenotype, we also collected and kept newly eclosed flies at 18^o^C for 5-7 days before preparing them for qPCR analysis. *Tdc2-GAL4* and the UAS lines were all crossed to the *white* (*w*) allele *w^1118^* and the heterozygous progeny were used as controls. The *w^1118^* allele was used because we set up our experimental crosses in such a way so the F1 males would be in a *w* mutant background. The level of *TfAP-2* and *Twz* expression in *Tdc2-GAL4* heterozygous controls were set as 100%, represented as 1 on the graph (Figure S1A). Compared with *Tdc2-GAL4* heterozygous controls (SE ± 0.05), *TfAP-2^RNAi2^* males kept at 29^o^C had only 0.23-fold (SE ± 0.05, P < 0.005) of normal *TfAP-2* expression levels (Figure S1A). On the other hand, *TfAP-2^RNAi2^* males maintained at 18^o^C had 0.92-fold (SE ± 0.07, P = 0.353) of normal *TfAP-2* expression (*Tdc2-GAL4^+/-^* 18^o^C SE ± 0.04) (Figure S1B). Similar to our previous publication using *Twz^RNAi1^*, expressing *Twz^RNAi2^* with the *Tdc2-GAL4* driver affected both *TfAP-2* and *Twz* expression. *Twz^RNAi2^* males raised at 29^o^C had only 0.28-fold (SE ± 0.09, P < 0.005) of the normal *Twz* RNA expression levels (Figure S1A). Raising *Twz^RNAi2^* males at 18^o^C had no significant affect on *Twz* expression levels compared to controls (Figure S1B). Interestingly, knocking down *Twz* suppressed *TfAP-2* expression levels, *Twz^RNA21^* males had only 0.20-fold (SE ± 0.09, P < 0.005) of normal *TfAP-2* expression (Figure S1A). Due to this result, for the rest of the manuscript when we refer to *Twz* knockdown males we actually mean *Twz* and *Tfap-2* double knockdowns, whereas when we refer to *Tfap-2* knockdowns we mean flies where only *Tfap-2* transcript levels were lowered.

***Dsk RNAi line functional***

To determine that the UAS-DskRNAi line was functioning properly we crossed the UAS-DskRNAi flies to the Dsk-GAL4 driver {Chen, 2012 #3337}, and performed quantitative RT-PCR (qPCR) to measure Dsk expression (Figure S1C). The level of Dsk expression in Dsk-GAL4 heterozygous controls was set as 100%, represented as 1 on the graph. Compared to controls, the Dsk-GAL4;UAS-DskRNAi flies had significantly less Dsk transcript (0.32-fold, SE ± 0,06, P < 0.005) (Figure S1C).
